# Food Order and Timing Effects on Glycaemic and Satiety Responses to Partial Fruit-for-Cereal Carbohydrate Exchange: A Randomized Cross-Over Human Intervention Study

**DOI:** 10.3390/nu15143269

**Published:** 2023-07-24

**Authors:** Suman Mishra, Andrew McLaughlin, John Monro

**Affiliations:** 1 The New Zealand Institute for Plant and Food Research Limited, Private Bag 11600, Palmerston North 4442, New Zealand; suman.mishra@plantandfood.co.nz (S.M.); andrew.mclaughlin@plantandfood.co.nz (A.M.); 2 Riddet Institute, University Avenue, Fitzherbert, Palmerston North 4474, New Zealand

**Keywords:** appetite, glycaemic response, carbohydrate exchange, kiwifruit, cereal

## Abstract

Postprandial glycaemic response amplitude plays a critical role in diabetic complications, but is subject to food order and temporal separation within a meal. Effects of partial fruit-for-cereal carbohydrate exchange on glycaemic and appetite responses, as affected by food order and separation, were examined using kiwifruit (KF) and wheaten breakfast cereal biscuit (WB). In a randomized cross-over intervention study, 20 subjects ingested 51.7 g of available carbohydrate as 74 g WB alone, or as 200 g KF and 37 g WB, each delivering 25.85 g of available carbohydrate. The 200 g KF was partially exchanged for 37 g of WB, at 90 min and 30 min before, at the same time as, or 30 min after, ingesting WB. Incremental satiety responses were derived from appetite scores measured using a visual analogue scale, and capillary blood glucose responses were monitored. In all exchanges, KF reduced the glycaemic response (iAUC) by 20–30% with no loss of total satiation. The incremental glycaemic and satiety responses to food ingestion followed each other closely. Glycaemic response amplitudes were reduced almost 50% compared with 74 g WB when KF ingestion preceded WB ingestion by 30 min, and less when the KF was ingested with or 30 min after the cereal. The results suggest that fruit most effectively suppresses the digestion of cereal carbohydrates if ingested long enough before the cereal to prevent overlap of the glycaemic responses, but close enough for fruit components that impede carbohydrate digestion or uptake to interact with the ingested cereal in the gut. Ethics approval was obtained from the Human and Disabilities Ethics Committee (HDEC) of the New Zealand Ministry of Health. The trial was registered with the Australian New Zealand Clinical Trials Registry (Trial ID: ACTRN12615000744550).

## 1. Introduction

Increased consumption of fruit has been recommended in the diets of people at risk of diabetes, partly because of a range of possible benefits arising from gut-level and systemic action of various fruit components [1], and also because fruit is also generally recognized as an essential component of a healthy diet. However, fruit has often suffered from consumer resistance because of the perception of it as a high carbohydrate food associated with risk of prediabetes, diabetes and diabetes complications, even though there is little direct evidence that moderate intakes of sugar have a causative role in glucose intolerance [2].

Our research on carbohydrate exchanges has shown that equi-carbohydrate partial substitution of breakfast cereal by kiwifruit reduced blood glucose peak height, delayed the onset of postprandial reactive hypoglycaemia, and maintained satiety. The kiwifruit therefore appeared to improve homeostatic blood glucose control in response to the meal. The antiglycaemic effect of fruit has been corroborated in other recent research [3].

The antiglycaemic effect of fruit during the carbohydrate exchange of cereal can be partly explained by the substitution of relatively low glycaemic index fruit sugars (including fructose, GI = 21) for starch (GI = 70 in wheaten biscuit). Other fruit factors, shown in a human intervention study to include dietary fibre and organic acids [4], may also play a role. The findings were consistent with in vitro studies that had indicated that dispersed cell wall remnants of kiwifruit could retard gut-level physical processes involved in glycaemic response, including digestion, mixing and diffusion [5]. With its high organic acid content, kiwifruit flesh has a strong buffering capacity and a low pH possibly reducing gastric salivary amylase activity and gastric emptying, leading to a further reduction in glycaemic response [6]. Thus, a combination of properties and effects could contribute to the ability of fruit to reduce glycaemic response to co-consumed cereal starch.

Because of the different mechanisms by which fruit might reduce digestion and absorption of carbohydrates, it is likely that the combination of gut processes that reduce glycaemic response will change with time after a meal. Therefore, the temporal separation of fruit and cereal intake in a meal could significantly modulate any antiglycaemic effect of the fruit. The impact of fruit organic acids may be immediate if their low pH inhibits gastric salivary amylase activity [6]. Longer term effects of organic acids mediated by the enterogastric reflex, in response to buffered low pH gastric contents entering the small intestine, may retard gastric emptying [7], further delaying glucose absorption. Dispersed fruit cell wall remnants may affect digestion and absorption of cereal starch throughout the gastrointestinal tract [8], as long as the fruit cell wall residues and cereals can interact in the gut. Systemic effects of prior fruit ingestion on glycaemic response to cereal may also occur if fruit sugars and other components pre-activate the hormonal and biochemical drivers of glucose homeostasis before cereal carbohydrate digestion [9]. Beyond the small intestine, colonic loading of fruit cell wall residues several hours after ingestion may inhibit foregut processes through distal feedback in a colonic brake mechanism, or second meal effect, activated by short-chain fatty acids from colonic fermentation of the fruit cell walls [10].

Another aspect of glucose homeostasis that has been linked to the insulin response to blood glucose is appetite, and its converse, satiety [11]. Satiety is the important motivational mechanism that regulates ingestion and is governed by a range of hormonal and neuroendocrine factors. It is important that the appetite effects of prescriptive interventions to reduce blood glucose are determined, because, in practice, increased motivation to eat is a challenge for interventions to reduce blood glucose.

The above considerations raise the possibility that the glycaemic benefits of fruit substitution of cereal starch in a meal, by partial carbohydrate exchange, may depend on the timing of fruit ingestion relative to the cereal ingestion. Recent studies have shown that the order in which foods are consumed in a meal affects the glycaemic response and appetite and conclude that the ingestion of foods high in available carbohydrates should be concentrated towards the end of a meal [12,13]. Another study suggested eating vegetables before carbohydrates was more effective than carbohydrate exchange in reducing glycaemia [14]. However, none of the foregoing studies included fruit. In the work described in the present paper, we focused specifically on the substitution of one type of carbohydrate food (fruit) for another (cooked cereal) to explore the way in which food order and temporal separation may be used to allow the health benefits of fruit to be obtained in a carbohydrate-containing meal while minimizing glycaemic cost. We provided participants with a meal of a highly glycaemic wheaten biscuit (WB) consisting mainly of digestible starch, ingested at a fixed time (breakfast), but partially substituted by kiwifruit (KF) using equi-carbohydrate exchange at various times around the cereal intake. The main outcome variables of the study were blood glucose response and satiety. We hypothesized that the time of ingestion of kiwifruit relative to cereal would significantly affect the influence of the carbohydrate exchange on the glycaemic and satiety responses to the breakfast cereal.

## 2. Methods

### 2.1. Meal Components

The kiwifruit (KF) used was *Actinidia chinensis* var. *chinensis* ‘Zesy002’ (marketed as Zespri™ SunGold™ Kiwifruit) were provided by Zespri Group Limited, Tauranga, New Zealand, in a ready-to-eat state of ripeness and processed within a few days of receipt. They were peeled, halved and snap frozen (−20 °C). The frozen fruit were allowed to thaw partially and were then crushed to a coarse pulp by briefly (10 s) chopping in a Halde food processor. The pulp was then divided accurately into individual 200 g portions and stored frozen within capped plastic containers until required.

A typical nutrient analysis of Zespri™ SunGold™ Kiwifruit is: protein 1.02%, fat 0.26%, available carbohydrate 12.4%, sugars 12.3% of which fructose is 5.8% and glucose 5.3%, dietary fibre 1.4%, energy 238 kJ/100 g (New Zealand Food Composition Database, www.foodcomposition.co.nz, accessed on 14 March 2023).

The breakfast cereal (WB) was Weet-Bix™ (Sanitarium, Auckland, New Zealand), purchased from a local supermarket. It is made of cooked wheat flakes compressed into a biscuit that disintegrates rapidly on wetting and consists mainly of rapidly digestible starch with little sugar. (Nutrient information: protein 12% *w*/*w*, fat 1.4% *w*/*w*, carbohydrate 67% *w*/*w* of which sugars make up 2.8%, dietary fibre 10.5%, energy 1470 kJ/100 g).

### 2.2. Analyses

#### Available Carbohydrates in Kiwifruit and Meals

The available carbohydrate content of the meals was measured by digesting the carbohydrates to reducing sugars with a combination of pancreatic amylase (P-7545, Sigma-Aldrich, Darmstadt, Germany), amyloglucosidase (E-AMG, Megazyme, Bray, Ireland) and invertase at 37 °C for 120 min.

### 2.3. Formulation of Meals

The carbohydrate analyses of the kiwifruit used showed that 200 g KF contains 25.85 g of available carbohydrates. As WB are 69.8% of available carbohydrates, 37 g of WB was equivalent to 25.85 g of available carbohydrates, and a fifty percent equi-carbohydrate substitution of WB by KF would require 200 g KF to replace 37 g of WB in a WB meal of 74 g, maintaining a total available carbohydrate intake of 51.7 g.

On that basis, the experimental meals were as summarised in Table 1: Meal 1 was the breakfast cereal alone (reference) for comparison with treatments 2–5. Meals 2–5 were to test the effect of proximity of KF ingestion to WB ingestion during carbohydrate exchange, on glycaemic and appetite responses, with all meals containing 51.7 g of available carbohydrates. Meals 6 and 7 were to allow the effects of splitting WB and KF meals, respectively, into to two equal portions of 25.85 g available carbohydrate, to allow the effect of meal splitting without changing meal composition to be determined, for both KF and WB. Meal 8 was compared with meal 1 to detect a possible second meal (colonic) effect of kiwifruit consumed on the prior evening, on response to 74 g WB.

Based on food composition database values, the energy and dietary fibre contents of the WB74 meal and the combinations of WB and KF in meals 2–5 (Table 1) were similar, although protein content was reduced in combinations containing KF (Table 2). The meals differed mainly in the type of available carbohydrate they contained, with nearly all the KF available carbohydrate in the form of fruit sugars, while very little of the starchy cereal carbohydrate was free sugar. The formulation of the meals for the present study was based on in-house analysis of SunGold kiwifruit (12.93 g available carbohydrate per 100 g) not on the Food composition database values (12.4 g/100 g), which were used to illustrate approximate differences in major components (Table 2).

### 2.4. Human Intervention Study

The human intervention study was approved by the Health and Disabilities Ethics Committee of the New Zealand Ministry of Health (15/CEN/72), and the trial was registered with the Australia New Zealand Clinical Trials Registry (Trial ID: ACTRN12615000744550).

The trial was run as a non-blinded randomized repeated measures study. All participants received all eight treatments in random order. It was not possible to blind the participants to the meals they were consuming.

#### 2.4.1. Recruitment and Screening

Volunteers were recruited using a flyer that briefly described the study. The initial appointment was made with the volunteers for pre-screening. The volunteers were asked initial recruitment questions to determine their suitability to take part in the study. The nature of the study and their involvement and responsibilities were described to them. Each volunteer was presented with an information sheet, containing study details, and an informed consent form. Volunteers who were willing to participate were asked to complete the General Health Questionnaire (GHQ) and had a capillary blood glucose test to check that their blood glucose and HbA1c were in the normal range (fasting blood glucose < 6.0 mmol/L, HbA1c < 40 mmol/mol). This was also to familiarise them with the blood sampling procedure to be used in the study should they volunteer to take part. Exclusion criteria included recent ill-health as gauged by the GHQ or self-report, known intolerance of kiwifruit or wheat products, and glucose intolerance as indicated by the fasting blood glucose and HbA1c in the preliminary test. All participants signed the informed consent form before participating in the study.

#### 2.4.2. Participants

**Figure A1:**
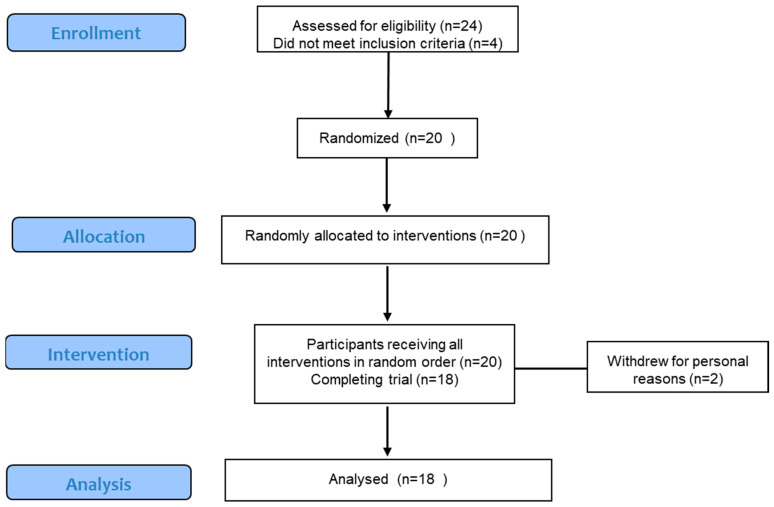
CONSORT diagram for the study of effects of order and temporal separation of fruit and cereal in a meal on glycaemic response and appetite.

The participant number for the trial was based on subject numbers in our previous clinical studies of kiwifruit and Weet-Bix interactions that had given highly significant results [5,6]. From a total of 24 respondents, 20 met the inclusion/exclusion criteria, from which 20 were randomly selected to take part in the trial, and 18 completed the trial (CONSORT diagram Appendix A Figure A1). Participants were male (n = 11) and female (n = 9), between the ages of 24 and 65 years (mean 44 years), with a BMI range of 18–35 kg/m^2^ (mean 27.2 kg/m^2^) and a fasting blood glucose range of 3.75–5.5 mmol/L (mean 4.5 mmol/L).

#### 2.4.3. Preparation and Ingestion of Meals

The kiwifruit component of the meals, frozen in 200 g portions, were thawed in a microwave immediately before consuming. The meals containing both WB and KF were well mixed immediately before serving and were ingested from a bowl with a spoon in less than 10 min. In the meals not containing kiwifruit water equivalent in volume to that in the kiwifruit was provided (84 mL per 100 g of kiwifruit) to maintain an approximately equal intake volume in all meals. In meals in which the intakes of WB and KF were separated the WB was ingested with water equivalent to that in 100 g of kiwifruit. The subjects in all trials were provided with an additional 200 mL of water to be sipped as dictated by thirst over the course of the trial.

#### 2.4.4. Participant Instructions

In preparation for each testing session, participants were asked to fast from 10.00 p.m. the night before a test without restricting water intake, to consume a similar evening (carbohydrate based) meal the night before each test session, and on the day of test avoid strenuous physical activity, smoking or consuming alcohol before testing. They were asked to be present at 08.30 h for the dietary intervention.

#### 2.4.5. Blood Glucose Measurement

On each test day, the volunteers were seated and asked to remain so for the duration of the test. Once the participant was relaxed and comfortable, a baseline blood sugar measurement was taken in duplicate. The participant was then given a test food and instructed to consume the whole amount within a ten-minute period, after which blood samples were collected at 15 min intervals in the first hour and thereafter at 30 min or 60 min intervals to a maximum of 210 min. Samples were thus collected at 0 (baseline × 2), 15, 30, 45, 60, 90, 120, 150 and 210 min depending on the treatment (Table 3). Blood glucose was measured using a HemoCue^®^ (Ängelholm, Sweden) blood glucose meter. Blood glucose concentrations were measured by finger-prick analysis of capillary blood.

#### 2.4.6. Satiety

Immediately before food consumption on the morning of the test, and for a maximum of 3.5 h after WB consumption, the subjects were asked to rate their level of satiety with a widely used and researched visual analogue scale for measuring dimensions of appetite [15]. The satiety measurement coincided with most, but not all the blood glucose samplings (Table 3).

The subjects were asked to rate their appetite at regular intervals coinciding with blood glucose measurements, using a four-dimension, 10-cm, visual analogue scale (VAS), with the dimensions: How hungry do you feel? (Not at all hungry—extremely hungry); How full do you feel? (Not at all full—extremely full); How strong is your desire to eat? (Not at all strong—extremely strong); How much food do you think you can eat? (Nothing at all—a large amount), based on published research on VAS scales for assessing appetite [15].

To obtain a satiety score, the four appetite dimensions were combined by averaging the four scores at each time. For all treatments, the scores on the different appetite dimensions tracked each other so closely (after subtracting the “How full do you feel?” value from 10), that it allowed the four dimensions within each treatment to be averaged. This average appetite score was converted to a satiety score by subtracting the mean appetite score at each time from the mean score at time 0, assumed to be the time of least satiety after the overnight fast and no breakfast. The mean appetite responses were converted to incremental satiety scores for subjects with an initial (time 0) appetite score of 5 or more. Individuals with mean appetite scores of <5 were excluded because of their low potential to show satiety effects at least at the start of a testing session.

### 2.5. Data Analysis

**Figure 1:**
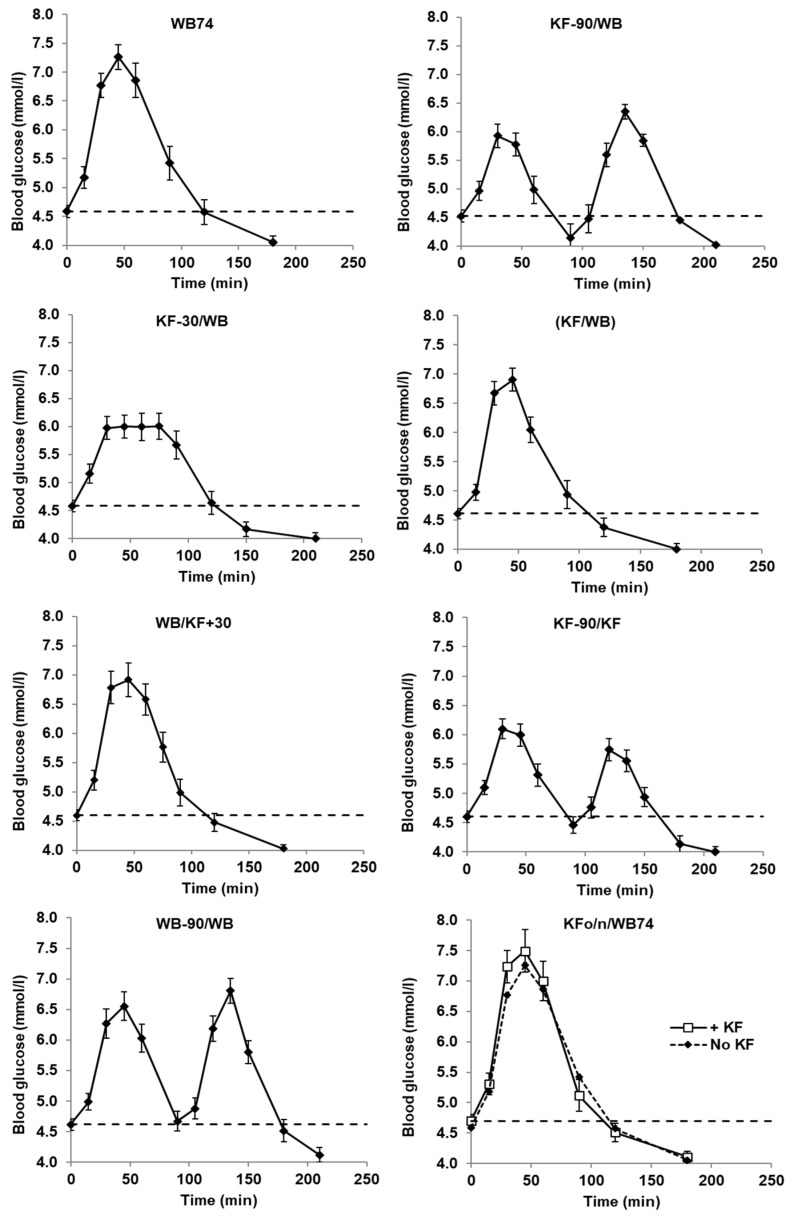
Blood glucose concentrations with time after ingesting flaked wheat biscuit (WB) alone or partially substituted by kiwifruit (KF). In all treatments, total available carbohydrate intake was 51.7 g, and in treatments in which KF was partially exchanged for WB, the WB and KF components each contributed 25.85 g. The dashed line is the Time 0 baseline. The treatments are described in Table 1. Means ± sem.

**Figure 2:**
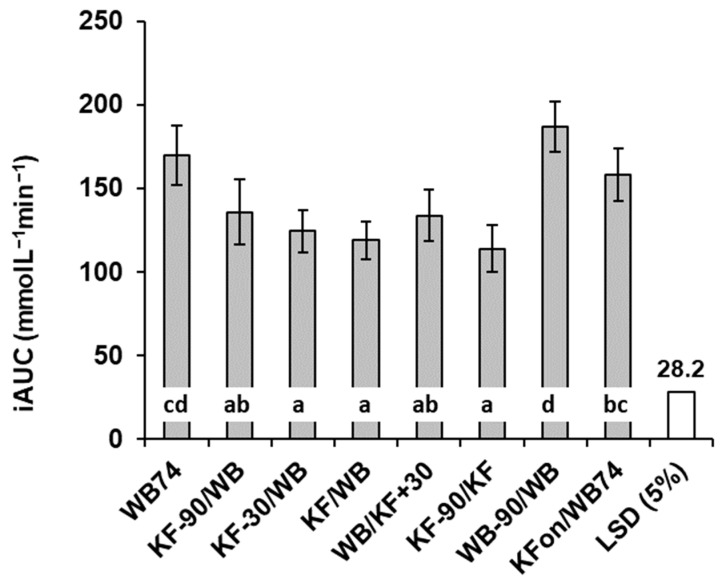
Total incremental area under the blood glucose response curves in response to kiwifruit (KF) and flaked wheat biscuit. Responses to meal types differed significantly (*p* < 0.001). Meals are summarized in Table 1. LSD = least significant difference (unfilled bar in graph). Means ± sem. Means with a letter in common do not differ significantly.

**Figure 3:**
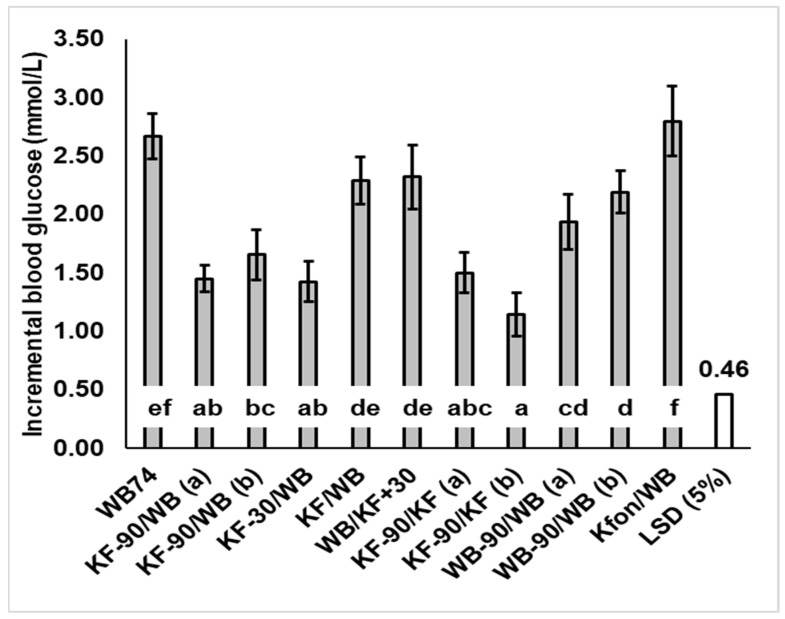
Mean peak glucose concentration increase over baseline (response amplitude) induced by kiwifruit (KF) and flaked wheat biscuit (WB) alone and in various combinations. Where a treatment caused two glycaemic response peaks, they are labelled (a) and (b). Peak blood glucose response differed significantly between meals (*p* < 0.001). LSD = least significant difference (5%) (unfilled bar in graph). Means ± sem. Means with a letter in common do not differ significantly. Values and % change from WB74 are given in Appendix A Table A1.

Mean blood glucose concentrations with time were plotted (Figure 1) and incremental blood glucose responses were calculated by subtracting each individual’s baseline value from subsequent measurements; these were then used to determine the positive incremental area under the curve (IAUC) for each individual by trapezoid summation, ignoring the areas under the baseline (Figure 2). Incremental peak responses were determined from the highest points on the mean blood glucose response curves of Figure 1 (Figure 3), and also as the mean of individual blood glucose peaks, irrespective of the time of occurrence (Appendix A Table A1).

The values are given in Appendix A Table A1.

In all analyses, time zero was taken as the start of the first ingestion of food (Table 3).

Data were entered into a Microsoft^®^ Excel spreadsheet for preliminary analysis and subsequently subjected to statistical analysis using Genstat software (version 22.1, VSN International Ltd., Hemel Hempstead, UK) using a linear mixed model with Subject as a random term and Meal type as a fixed term. Post hoc comparisons among means were made using Fisher’s least significant differences at *p* = 0.05 (5% LSD).

## 3. Results

### 3.1. Carbohydrate Analysis

Carbohydrate analyses showed that KF contained 12.93 ± 0.4 g of available carbohydrate per 100 g. The WB breakfast cereal was 69.8% available carbohydrate. From these results, it was possible to calculate the proportions of KF (200 g) and WB (37 g) to be included in the meals (as shown in the Methods Section).

### 3.2. Glycaemic Response

The mean blood glucose responses to all meals commenced at a baseline value of 4.6 and all had returned to a value of about 4.0 mmol/L by the end of each trial, but between these points there were considerable differences between the meals (Figure 1). The timing of kiwifruit ingestion relative to WB ingestion had a distinct influence on the characteristics of the incremental blood glucose responses (Figure 2). All meals involving equi-carbohydrate substitution of WB by KF (meals 2–5) showed a significant reduction in glycaemic response measured as incremental areas under the blood glucose response curves (iAUC) compared with the unsubstituted WB (74 g) controls (Figure 2). However, the combined iAUCs for each treatment containing 200 g KF and 37 g WB (meals 2–5) were not significantly different from one another, but they were significantly less than the iAUCs for the 74 g WB treatment, which contained the same amount of available carbohydrate. Therefore, KF was capable of reducing exposure to glycaemia by equi-carbohydrate substitution at any time within the 120 min time band tested from 90 min before to 30 min after WB ingestion.

The timing of KF consumption did, however, have a substantial and significant (*p* < 0.001) effect on the intensity of the glycaemic response, gauged by the height of the postprandial peak (Figure 3). Peak heights when KF was consumed up to 30 min before WB were much lower than when KF was consumed at the same time as or 30 min after the WB. Incremental peak heights on the curves of Figure 1 (Figure 3), and the mean of individual peak heights for each treatment irrespective of time (Appendix A Table A1), followed a similar pattern, although the time-independent values were slightly higher.

Of the 37 g WB/200 g KF combinations (treatments 2–5), the WB/KFSG+30 treatment showed the greatest peak height, presumably because the KF was added after the rapid digestion of WB had occurred. Therefore, the KF fibre and remnants could not moderate the glucose absorption from WB, and the response to KF would have added to the WB response (Appendix A Figure A5). On the other hand, in the case of the SG-30/WB treatment, the blood glucose loading from the second intake (WB) probably overlapped sufficiently with that of the first meal for the insulin response to the first meal (200 g KF) to reduce the glycaemic response to the second meal. Speaking generally, the results suggest the greatest peak reductions will be achieved when kiwifruit is consumed close enough to a glycaemic food for the properties of its digestion-resistant remnants, or the homeostatic response to it, to have a moderating influence, but far enough apart for the glycaemic responses not to coincide enough to have an additive effect.

The same meal components were fed as two intakes 90 min apart (KF-90/KF and WB-90/WB) to see if an effect of meal splitting into two intakes had a consistent effect on blood glucose response amplitude. In the case of KF the effect of the second KF intake was less than the first by 22.5%, whereas there was no difference between the two WB intakes (Figure 3; KF-90/KF versus WB-90/WB).

Ingesting 200 g of KF the evening before WB74 (treatment KFon/WB74) had no effect on the response to WB74, which was remarkably similar to WB74 alone (Figure 1). There was no second meal effect in the overnight time interval and with the food quantities tested.

### 3.3. Satiety

**Figure A2:**
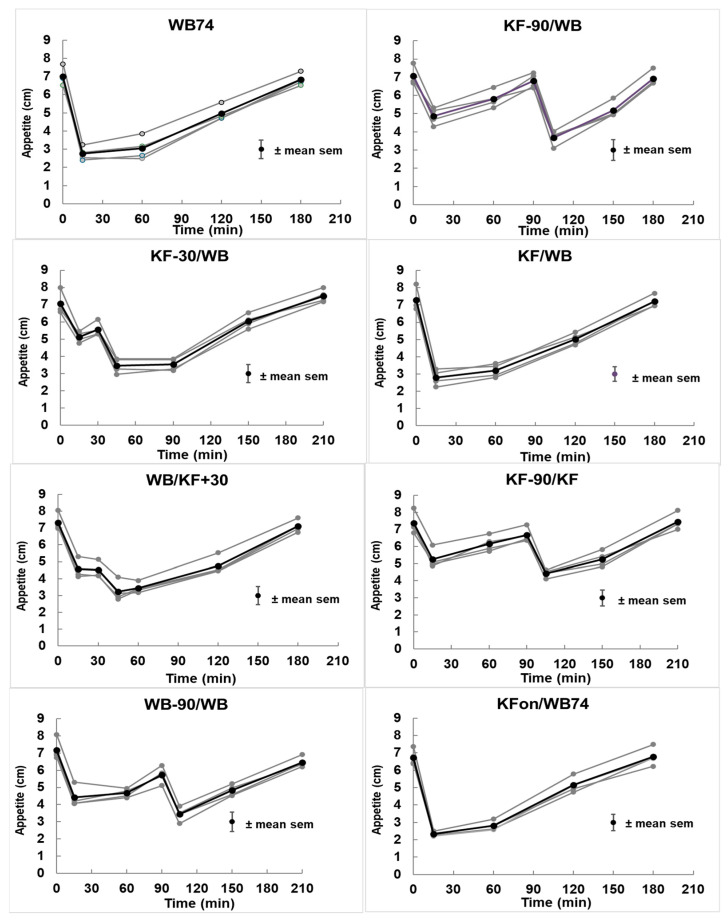
Changes in the four dimensions of appetite (all in grey) and their mean (bold black) plotted against time. The treatments are described in Table 1. For clarity, only the mean of the sem for all points and dimensions in a treatment is shown, and the four dimensions of appetite are so closely superimposed and similar in profile that there was no benefit in identifying them separately.

The responses along all four dimensions of appetite were remarkably consistent within treatments and between similar treatments. Within all treatments, the four dimensions of appetite tracked each other very closely even when not perfectly superimposed (Figure A2). The within-treatment reproducibility of the dimensions justified averaging them to create a single graph of appetite for each treatment (bolded curves in Figure A2). The consistency between treatments can be seen by comparing treatments 1 (WB74) with 8 (KFon/WB74), or treatment 2 (KF-90/WB) with 6 (WB-90/WB) for the first 90 min.

**Figure 4:**
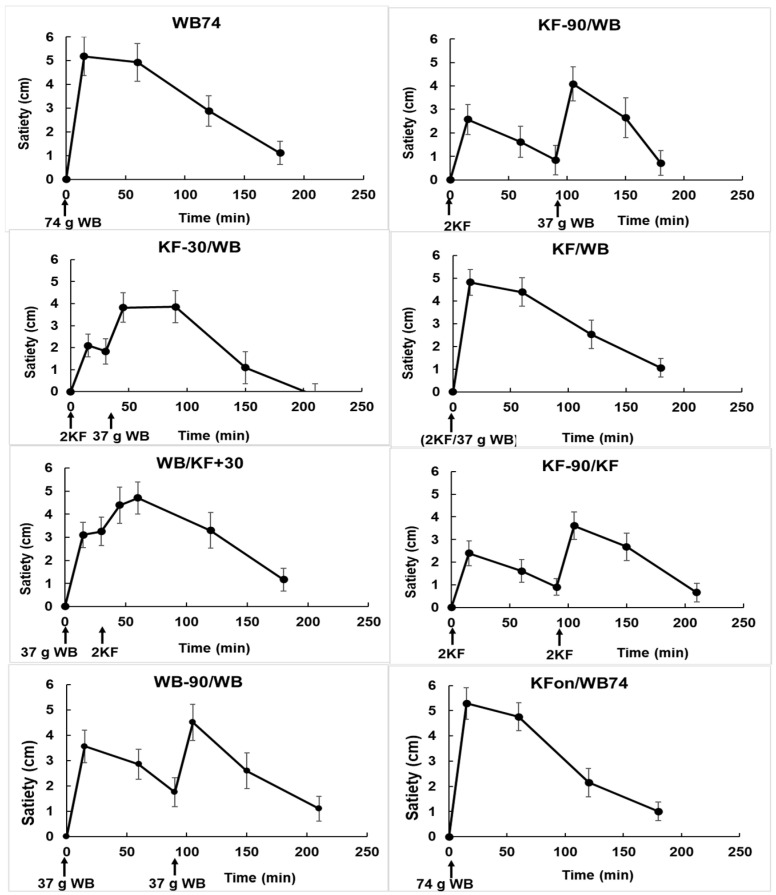
Changes in satiety with time in response to the ingestion of kiwifruit (KF) and flaked wheat biscuit (WB) in the various combinations described in Table 1. The times of ingestion of the KF and WB components of the meals are shown along the X-axis. Means ± sem.

**Figure A3:**
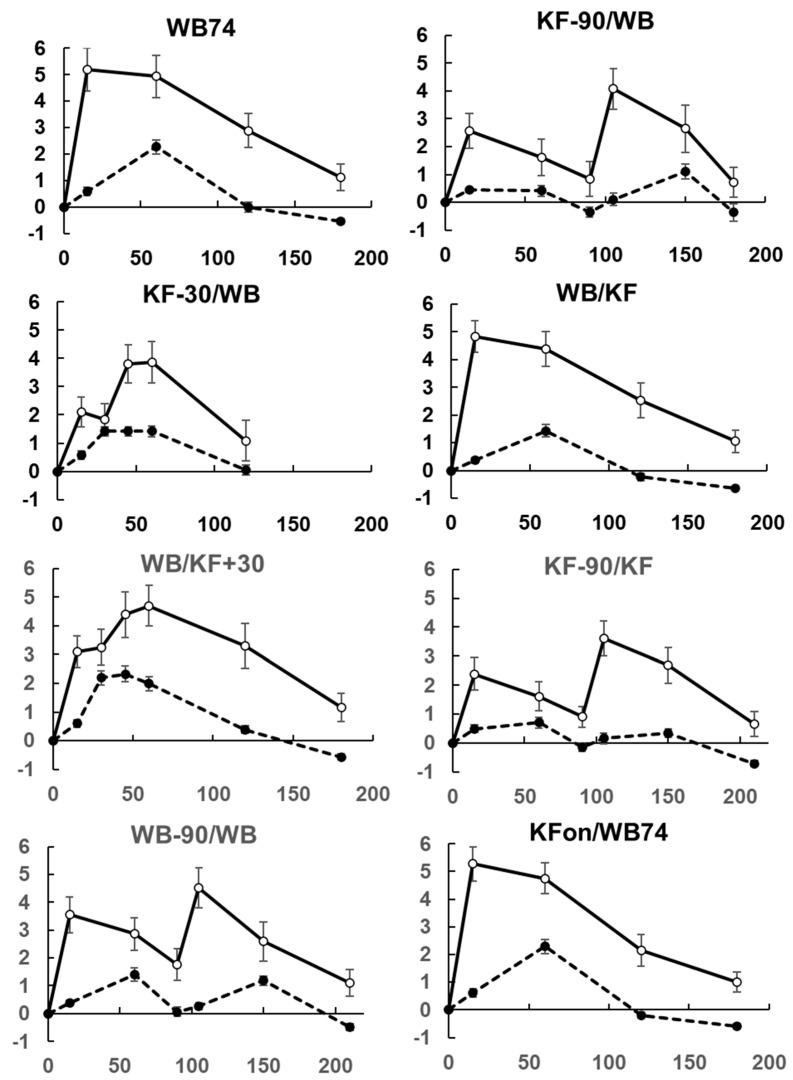
Correspondence between increments in blood glucose concentration (dotted line, mmol/L) and satiety (solid line, cm). Means ± sem.

Incremental satiety scores (Figure 4) calculated by subtracting the mean appetite scores at each time from the mean score at time zero appeared to be reproducible (compare treatments 1 and 8). Their approximate correspondence with the blood glucose response curves (Figure 1, Appendix A Figure A3) suggests that they are valid, as both appetite and blood glucose changed similarly in response to food intake, with the blood glucose response being slightly less immediate.

**Figure 5:**
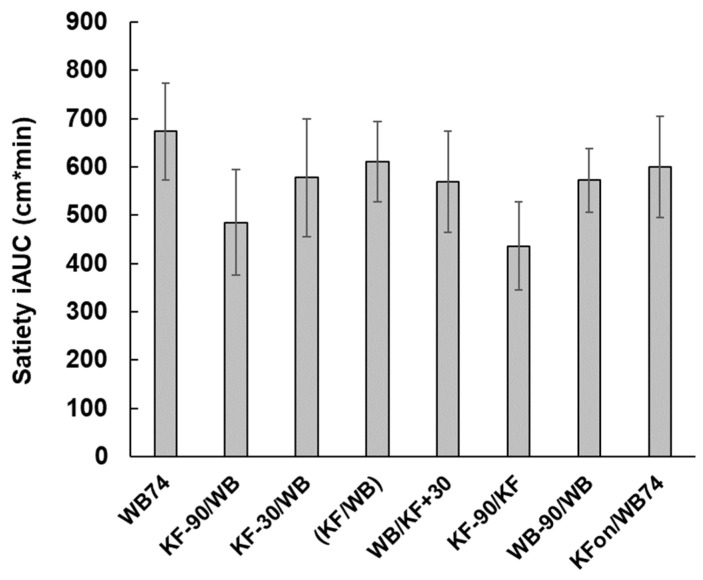
Area under the satiety curves (Figure 4) for the treatments described in Table 1. Mean incremental satiety did not differ among meal types (*p* = 0.65). Mean ± sem. The values are given in Table A1.

From the areas under the incremental satiety scores (Figure 5) the KF appeared to be slightly less satiating than WB (compare treatments 6 (KF-90/KF) and 7 (WB-90/WB)), although the difference was not significant. When KF was ingested within ± 30 min of WB (treatments 3, 4, 5), the areas under the incremental satiety curves were similar to those of treatments 1 and 8 (both WB74).

## 4. Discussion

Partial equi-carbohydrate exchange of KF for WB at any time within the 120 min time band tested, from 90 min before to 30 min after WB ingestion (treatments 2–5, Table 1), reduced the glycaemic response (iAUC) compared with the WB-only reference (treatment 1). The effect of KF substitution of WB will be partly due to fructose in fruit sugar replacing rapidly digested starch in WB, as fructose, with a glycaemic index of 21, has much less impact on blood glucose than starch in WB, which has a GI of 70. The effects of KF in response to WB in the different treatments may also be explained in terms of the time-course of the blood glucose response to carbohydrate in the foods, and the duration of effects of organic acids and fibre in the kiwifruit on the processes involved in starch digestion and absorption. In previous research, we have shown that digested kiwifruit remnants, predominantly cell wall material, may retard physical processes of digestion such as mixing and diffusion [5]. Kiwifruit also contain a high concentration of organic acids with a low pH (about pH 3.4) and a high buffering capacity. In vivo studies showed that both fibre and organic acids in kiwifruit contributed to a reduced glycaemic response to co-consumed WB [4].

In terms of energy and dietary fibre content the treatments involving WB and KF (2–5) were similar to the WB74 treatment, although they differed in protein content (Table 2). In metabolic terms, the meals may have differed because of the differences in the type of available carbohydrates that they contained. Available carbohydrate in KF was almost entirely fruit sugar, which is approximately 50% fructose in KF, whereas available carbohydrates in WB are mainly rapidly available starch. Also, the cereal dietary fibre in WB will have differed considerably from the dietary fibre present in ripe kiwifruit in its physical properties, so the effects of the two forms of dietary fibre on digestive processes are likely to have differed.

The present results showed that any separation of intakes that resulted in separation of the glycaemic responses to each component reduced the peak height by preventing the addition of responses. And if the responses can be separated within the time period in which the KF fibre and organic acids can interact with cereal digestion to reduce glycaemic response, a further reduction could be expected.

**Figure A4:**
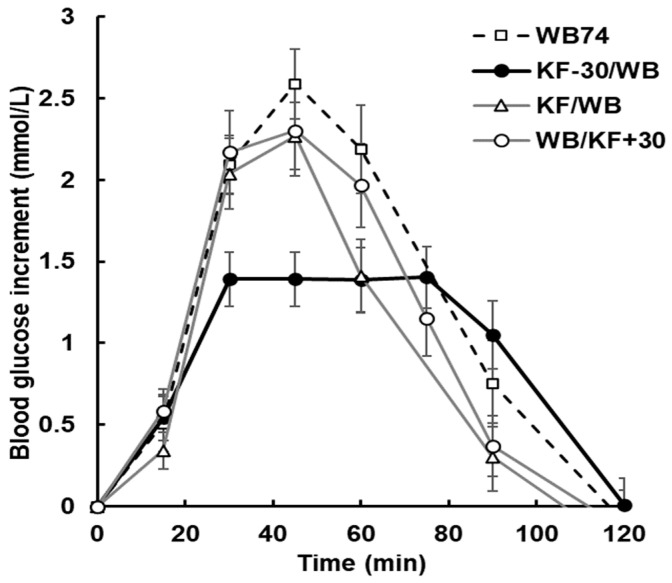
Glycaemic response to kiwifruit (KF) and wheat biscuit (WB), with KF ingested 30 min before wheat biscuit (WB) (treatment KF-30/WB; bold, black markers), at the same time as (KF/WB), or 30 min after WB (WB/KF+30), compared with wheat biscuit alone (WB74). All meals delivered 51.7 g of available carbohydrate. Order and timing have substantially reduced response amplitude in the KF-30/WB treatment. Treatments are summarized in Table 1. Means ± sem.

**Figure A5:**
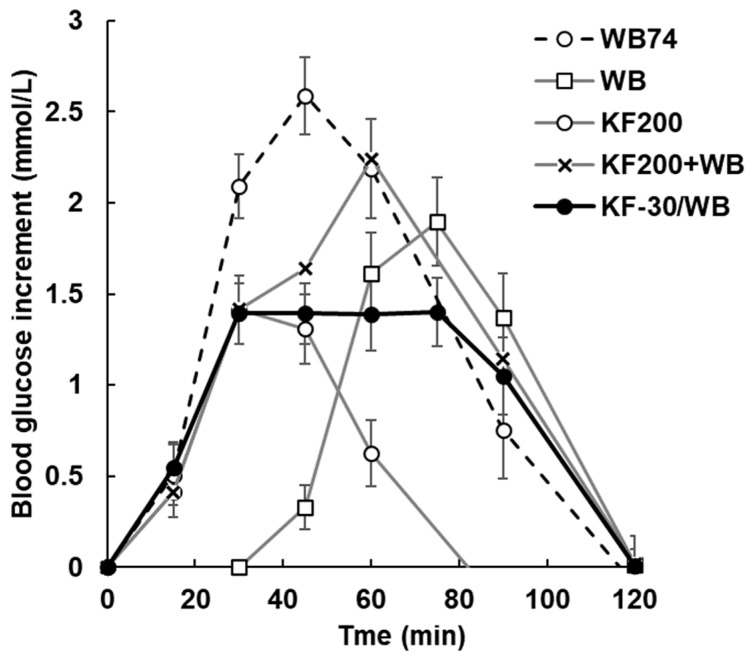
Blood glucose response to WB alone at an intake of 51.7 g of carbohydrate (WB74), to 37 g of wheaten biscuit (WB), to 200 g of kiwifruit (KF200), each delivering 25.85 g available carbohydrate, to the sum of the two (KF200+WB; theoretical), and to 200 g KF ingested 30 min before 37 g WB (KF-30/WB). WB starts at 30 min to coincide with its ingestion in the KF-30/WB treatment. KF has suppressed the response to WB in the KF-30/WB treatment compared with the response to WB alone. Treatments are summarized in Table 1. Means ± sem.

In the case of KF-90/WB (treatment 2), blood glucose concentrations, with the help of the insulin response, had returned to baseline after about 60 min, while most of the effects of organic acids and fibre on gastrointestinal processes affecting glucose absorption would have been completed by the time of WB ingestion 90 min after KF. Therefore, given that all meals had the same carbohydrate content, and the separation of WB and KF probably eliminated gastrointestinal effects of KF on WB digestion and absorption, the main reason that KF-90/WB induced a lower glycaemic response (iAUC) than WB 74 would probably have been the partial substitution of fruit sugars (containing fructose, GI 22) for starch (GI 70 in WB). When the KF was ingested as a 30 min preload to the WB (treatment 3, KF-30/WB), there were several factors that may have contributed to the substantially reduced response. Firstly, the partial substitution of fructose for glucose. Secondly, the KF organic acids and dietary fibre would have been present and active in the stomach before and from the time of ingestion of the WB, possibly leading to a suppression of salivary amylase activity in the stomach [6], and/or a delay in gastric emptying [7]. Thirdly, the glycaemic and insulin responses to the kiwifruit would have peaked and commenced its descent by the time the glycaemic response to WB had initiated (as in KF-90/KF curves, Figure 1). Therefore, the glycaemic response peaks to the KF and WB components did not overlap, resulting in a blunted, flat maximum response to the KF-30/WB treatment (Figure 1 and Appendix A Figure A4). Furthermore, the fact that the response to the combination was flat suggests that the KF component may have somewhat suppressed the WB response. Because both components contained the same carbohydrate content, but the WB component had a higher GI than the KF component it should, in theory, have given a greater response. This is made clear by superimposing the graphs of KF200, WB37, (KF200+WB37) and KF-30/WB (Appendix A Figure A5).

When the WB and KF were consumed together (KF/WB, treatment 4), the same factors as in the KF-30/WB treatment would have operated except that the glycaemic responses to the KF and WB available carbohydrate would have coincided. The amount of carbohydrate absorbed in both treatments was the same, except that the total carbohydrate load in the KF/WB treatment was ingested as a single load, and the response amplitude was correspondingly higher, without much difference in the total IAUC.

When the WB was ingested 30 min before the KF component, there would have been time for most of the glycaemic response to WB to have developed before any of the KF components, such as organic acids and dietary fibre, could have inhibited the digestion of WB starch and/or its delivery to the small intestine. Furthermore, as the cooked starch in WB has a greater intrinsic glycaemic potency than fruit sugars in KF, most of the potential glycaemic response to the combination of KF and WB would have developed free of KF inhibition.

A treatment involving the ingestion of 200 g of kiwifruit at 10 pm the evening before consuming 74 g of wheaten biscuit (Treatment 8, KFon/WB74) was included to test for a second meal effect within a manageable time frame. The gap between the ingestion of the 200 g KF and the 74 g of WB was 10 h. The glycaemic responses to WB74 with and without the kiwifruit were almost identical (treatments 1 and 8, Figure 1). Even though no effect of KF the night before was detected, the close correspondence of the two curves suggested, if nothing else, that the measurements were highly reproducible. As the second meal effect has been attributed to organic acid production by the colonic microbiota [10], it is possible that the 10 h time separation between ingestion of 200 g of KF and WB74 allowed colonic fermentation of the KF cell walls to be completed too far ahead of WB74 ingestion to have any effect on the glycaemic response to WB.

The temporal separation of fruit and cereal in the present study did not achieve large reductions in the total areas under the blood glucose response curve, but did achieve significant reductions in peak height. It is now considered that glycaemic response amplitude is an important factor in the damage caused by postprandial glycaemia, leading to diabetic complications through the related processes of glycation, oxidative stress, and inflammation [16]. The present study has shown that in a carbohydrate exchange approach to glycaemia management, the nutritional benefits of consuming kiwifruit or other fruit may be obtained while glycaemic response amplitude is reduced through the management of food timing and sequence, and satiety is maintained.

Although the total amount of satiety did not differ between treatments, all treatments had a satiating effect. The incremental satiety results suggest that if KF is to be used in a carbohydrate exchange format, consuming the KF at about 30 min before WB would have the benefit of combining the satiety effects of the two components while disengaging their peak glycaemic responses. Although it is not easy to translate the satiety scores directly into human behaviour, the satiety results (Figure 4) at least suggest that when using temporal separation of foods in carbohydrate exchange for control of glycaemia, the effects of delayed intake on satiety, seen in the treatments with a 90 min separation, should be considered as a factor in dietary management.

A possible criticism of using fruit for cereal exchange is that increased fruit intake leads to increased intake of fructose, which has been associated with harmful metabolic and health effects through products of its metabolism, such as uric acid, and through its reactivity in forming advanced glycation end products [17,18]. However, much of the evidence against fructose has come from studies using high fructose doses, and there is little evidence that fructose consumed in customary intakes of fruit would be harmful in an otherwise healthy diet [19].

A limitation of the present study was that the resources available limited the number of secondary outcomes, such as hormonal changes, that were measured. However, in a previous study on the interaction of WB and KF, we showed that the glycaemic response to both WB and KF, and combinations of the two, were followed very closely by insulin [4], with a correlation of R = 0.98. Further research using fewer food combinations and larger numbers of participants than in the present study should measure changes in various hormones governing glucose homeostasis and appetite, as objective physiological correlates to support the clear changes in both glycaemic response and appetite observed in the present study. Such studies would also benefit from more complete blinding than was possible in the present study, given the subjective nature of appetite scoring. The satiety results raise the interesting question—would the increase in satiety caused by consuming kiwifruit 30 min before cereal lead to a corresponding decrease in voluntary intake of cereal made available ad libitum? If so, changes in appetite due to the consumption of kiwifruit before a meal may naturally lead to an approximately equi-carbohydrate or equi-energetic food intake, without the need for prescriptive use of carbohydrate exchanges.

In terms of meal construction and dynamics, a 30 min separation of food components is not unreasonable and is certainly consistent with the suggestion that “slow food” is a healthier form of eating than consuming large meals quickly [20]. “Slow” meals provide the opportunity to plan food order to benefit from food and food component interactions that reduce glycaemic response, and to separate response peaks. It has been suggested that meat and vegetables eaten before carbohydrate in a meal reduce glycaemic response [12]. The results presented here suggest an alternative approach would be to split the carbohydrate into two intakes separated by about 30 min; a carbohydrate-rich entrée followed by a low carbohydrate main course and finishing with a carbohydrate-based desert. Such spreading of carbohydrate intake may also tend to moderate the demands on physiological systems for energy homeostasis, such as the insulin response, which closely follows glycaemic loading and blood glucose peak height.

There is more research required on the place and role of fruit in meals, especially on the influence of different types of food on the antiglycaemic effectiveness of fruit components. Perhaps not only the separation of responses, but also the separation of meal items could be used to reduce meal glycaemic potency.

## 5. Conclusions

The research presented here suggests that the effective use of fruit in a carbohydrate exchange format to reduce the postprandial glycaemic response to starchy food depends on:

- Equi-carbohydrate partial substitution of low GI fructose in fruit sugars for higher GI carbohydrates such as cooked starch.
- Separation of fruit and starchy foods in a meal enough to avoid overlap of the glycaemic responses to each.
- Ingestion of the fruit no later than the starchy food to ensure that the starch digestion is subjected to interactions with fruit remnants and components, such as dietary fibre and organic acids, that delay digestion and absorption.

## Figures and Tables

**Table 1:** Treatments in which separation of kiwifruit (KF) and flaked wheat biscuit (WB) ingestion was varied. Treatments 1–7 contained 51.7 g of available carbohydrate in either 74 g WB, or 200 g KF plus 37 g WB, or 400 g KF.

No.	ID	Description of Treatment
1	WB74	WB (74 g) ingested alone
2	KF-90/WB	KF (200 g) ingested 90 min before consuming 37 g WB
3	KF-30/WB	KF (200 g) ingested 30 min before consuming 37 g WB
4	KF/WB	KF (200 g) ingested at the same time as 37 g WB
5	WB/KF+30	KF (200 g) ingested 30 min after consuming 37 g WB
6	KF-90/KF	KF (200 g) ingested 90 min before another 200 g kiwifruit.
7	WB-90/WB	WB 37 g ingested 90 min before another 37 g WB (total 74 g)
8	KFon/WB74	WB (74 g) ingested the morning after having consumed 200 g KF at 22 h the previous evening

**Table 2:** Major components of meals in Table 1 estimated from food composition table values.

Meal	Energy (kJ)	Available Carbohydrate (g)	Sugars (g)	Dietary Fibre (g)	Protein (g)
74 g WB	1088	49.6	1.48	7.7	8.88
37 g WB + 200 g KF	1020	49.6	25.5	6.7	6.48
37 g WB	544	24.8	0.74	3.9	4.44
200 g KF	476	24.8	24.8	2.8	2.04
400 g KF	952	49.6	49.6	5.6	4.08

**Table 3:** Blood sampling times after initial food ingestion at time 0. Appetite was measured at the times underlined and in bold font.

	Meal	Time (min)												
1	WB74	**0**	**15**	30	45	**60**		90		**120**			**180**	
2	KF-90/WB	**0**	**15**	30	45	**60**		**90**	**105**	120	135	**150**	180	**210**
3	KF-30/WB	**0**	**15**	**30**	**45**	60	75	**90**		120		**150**		**210**
4	KF/WB	**0**	**15**	30	45	**60**		90		**120**			**180**	
5	WB/KF+30	**0**	**15**	**30**	**45**	**60**	75	90		**120**			**180**	
6	KF-90/KF	**0**	**15**	30	45	**60**		**90**	**105**	120	135	**150**	180	**210**
7	WB-90/WB	**0**	**15**	30	45	**60**		**90**	**105**	120	135	**150**	180	**210**
8	KFon/WB74	**0**	**15**	30	45	**60**		90		**120**			**180**	

## Data Availability

Data is available from the corresponding author.

## Appendix A

**Table A1:** Values for incremental areas under the curve (IAUC) of blood glucose responses (BGR IAUC, presented visually in Figure 1 and Figure 2), satiety responses (Satiety IAUC, visually in Figure 4), and the average of the blood glucose at highest time point of the blood glucose response curve (Figure 1) (Peak (a), visually in Figure 3), and the average of the highest individual responses to a treatment irrespective of time (Peak (b) not shown elsewhere).

	BGR IAUC	Satiety IAUC		Peak (a)	Peak (b)
	mmol/L/min	cm × min		mmol/L	mmol/L
WB74	169.6	644.4	WB74	2.67	2.992
KF-90/WB	135.6	485.0	KF-90/WB (a)	1.448	1.617
KF-30/WB	124.5	557.6	KF-90/WB (b)	1.653	2.097
(KF/WB)	118.9	595.9	KF-30/WB	1.425	1.985
WB/KF+30	133.7	557.3	(KF/WB)	2.29	2.465
KF-90/KF	113.9	426.3	WB/KF+30	2.32	2.76
WB-90/WB	186.7	531.1	KF-90/KF (a)	1.498	1.782
KFon/WB74	157.9	580.4	KF-90/KF (b)	1.143	1.377
			WB-90/WB (a)	1.935	2.265
			WB-90/WB (b)	2.19	2.275
			KFon/WB74	2.795	3.105
Average LSD	28.22	224		0.4602	0.3587

**Table A2:** Differences between maxima of the blood glucose response curves shown in Figure 1, and the percent reduction in peak height compared with WB74 for each curve. Where two peaks occur in response to a single treatment, they are labelled (a) and (b) in order of occurrence. Means with a letter in common to the right do not differ significantly.

Meal (Table 1)	Mean (mmol/L)		sem	Decrease from WB74 (%)
WB74	2.67	ef	0.19	0
KF-90/WB (a)	1.45	ab	0.11	46
KF-90/WB (b)	1.65	bc	0.22	38
KF-30/WB	1.43	ab	0.18	47
KF/WB	2.29	de	0.20	14
WB/KF+30	2.32	de	0.28	13
KF-90/KF (a)	1.50	abc	0.17	44
KF-90/KF (b)	1.14	a	0.19	57
WB-90/WB (a)	1.94	cd	0.24	28
WB-90/WB (b)	2.19	d	0.18	18
KFon/WB74	2.80	f	0.30	−5
LSD (5%)	0.460			
*p*	<0.001			

## References

[1] Dreher M.L. (2018). Whole Fruits and Fruit Fiber Emerging Health Effects. Nutrients.

[2] Hosseini F., Jayedi A., Khan T.A., Shab-Bidar S. (2022). Dietary carbohydrate and the risk of type 2 diabetes: An updated systematic review and dose-response meta-analysis of prospective cohort studies. Sci. Rep..

[3] Lu X.J., Lu J.C., Fan Z.H., Liu A.S., Zhao W.Q., Wu Y.X., Zhu R.X. (2021). Both Isocarbohydrate and Hypercarbohydrate Fruit Preloads Curbed Postprandial Glycemic Excursion in Healthy Subjects. Nutrients.

[4] Monro J., Mishra S., Stoklosinski H., Bentley-Hewitt K., Hedderley D., Dinnan H., Martell S. (2022). Dietary Fibre and Organic Acids in Kiwifruit Suppress Glycaemic Response Equally by Delaying Absorption-A Randomised Crossover Human Trial with Parallel Analysis of C-13-Acetate Uptake. Nutrients.

[5] Mishra S., Monro J. (2012). Kiwifruit remnants from digestion in vitro have functional attributes of potential importance to health. Food Chem..

[6] Freitas D., Le Feunteun S. (2018). Acid induced reduction of the glycaemic response to starch-rich foods: The salivary-amylase inhibition hypothesis. Food Funct..

[7] Hunt J.N., Knox M.T. (1969). Slowing of gastric emptying by 9 acids. J. Physiol..

[8] Grundy M.M.L., Edwards C.H., Mackie A.R., Gidley M.J., Butterworth P.J., Ellis P.R. (2016). Re-evaluation of the mechanisms of dietary fibre and implications for macronutrient bioaccessibility, digestion and postprandial metabolism. Br. J. Nutr..

[9] Dimitriadis G.D., Maratou E., Kountouri A., Board M., Lambadiari V. (2021). Regulation of Postabsorptive and Postprandial Glucose Metabolism by Insulin-Dependent and Insulin-Independent Mechanisms: An Integrative Approach. Nutrients.

[10] Brighenti F., Benini L., Del Rio D., Casiraghi C., Pellegrini N., Scazzina F., Jenkins D.J.A., Vantini I. (2006). Colonic fermentation of indigestible carbohydrates contributes to the second-meal effect. Am. J. Clin. Nutr..

[11] Flint A., Gregersen N.T., Gluud L.L., Moller B.K., Raben A., Tetens I., Verdich C., Astrup A. (2007). Associations between postprandial insulin and blood glucose responses, appetite sensations and energy intake in normal weight and overweight individuals: A meta-analysis of test meal studies. Br. J. Nutr..

[12] Shukla A.P., Mauer E., Igel L.I., Truong W., Casper A., Kumar R.B., Saunders K.H., Aronne L.J. (2018). Effect of Food Order on Ghrelin Suppression. Diabetes Care.

[13] Shukla A.P., Dickison M., Coughlin N., Karan A., Mauer E., Truong W., Casper A., Emiliano A.B., Kumar R.B., Saunders K.H. (2019). The impact of food order on postprandial glycaemic excursions in prediabetes. Diabetes Obes. Metab..

[14] Imai S., Matsuda M., Hasegawa G., Fukui M., Obayashi H., Ozasa N., Kajiyama S. (2011). A simple meal plan of ‘eating vegetables before carbohydrate’ was more effective for achieving glycemic control than an exchange-based meal plan in Japanese patients with type 2 diabetes. Asia Pac. J. Clin. Nutr..

[15] Flint A., Raben A., Blundell J.E., Astrup A. (2000). Reproducibility, power and validity of visual analogue scares in assessment of appetite sensations in single test meal studies. Int. J. Obes..

[16] Watt C., Sanchez-Rangel E., Hwang J.J. (2020). Glycemic Variability and CNS Inflammation: Reviewing the Connection. Nutrients.

[17] Gugliucci A. (2017). Formation of Fructose-Mediated Advanced Glycation End Products and Their Roles in Metabolic and Inflammatory Diseases. Adv. Nutr..

[18] Caliceti C., Calabria D., Roda A., Cicero A.F.G. (2017). Fructose Intake, Serum Uric Acid, and Cardiometabolic Disorders: A Critical Review. Nutrients.

[19] Laughlin M.R. (2014). Normal Roles for Dietary Fructose in Carbohydrate Metabolism. Nutrients.

[20] Hawton K., Ferriday D., Rogers P., Toner P., Brooks J., Holly J., Biernacka K., Hamilton-Shield J., Hinton E. (2019). Slow Down: Behavioural and Physiological Effects of Reducing Eating Rate. Nutrients.

